# Gray Matter Volumetry and Cognitive Functioning in Pediatric Posterior Fossa Tumor Survivors

**DOI:** 10.3390/cancers18030495

**Published:** 2026-02-02

**Authors:** Kristien Bullens, Charlotte Sleurs, Jeroen Blommaert, Karen Van Beek, Jurgen Lemiere, Sandra Jacobs

**Affiliations:** 1 Department of Oncology, KU Leuven, 3000 Leuven, Belgium; kristien.bullens@kuleuven.be (K.B.);; 2 Department of Cognitive Neuropsychology, Tilburg University, 5037 AB Tilburg, The Netherlands; 3 Department of Imaging and Pathology, Translational MRI, 3000 Leuven, Belgium; 4 Department of Radiation Oncology, PARTICLE Proton Therapy Centre University Hospital Leuven, 3000 Leuven, Belgium; 5 Department of Pediatric Hematology and Oncology, University Hospitals Leuven, 3000 Leuven, Belgium

**Keywords:** childhood brain tumors, infratentorial neoplasms, gray matter volumetry, cognition, radiotherapy, neuroimaging, long-term

## Abstract

**Background/Objectives**: Posterior fossa tumors (PFTs) often require radiotherapy which may damage both cortical and subcortical brain tissue. We examined voxel-based and region-based gray matter volume (GMV) alterations and explored their relation to cognitive functioning. **Methods**: Using T1-weighted MR imaging, intelligence scores from the Wechsler Adult Intelligence Scale-IV (WAIS-IV), and domain scores for language, learning, and memory, complex attention and cognitive flexibility were investigated in 18 of 21 assessed PFT survivors and 21 matched controls. GMV was quantified with voxel-based and region-based volumetry, compared among groups (controls versus survivors, irradiated versus non-irradiated survivors), and correlated with cognitive performance. **Results**: Survivors showed reduced GMV in two voxel-based clusters, located in the left occipital fusiform area and the left pallidum. Region-based analyses showed lower GMV in survivors located in subcortical areas, while higher GMV was observed in the left inferior temporal region. Survivors who received craniospinal radiation exhibited reductions in GMV in bilateral thalami, right ventral diencephalon, and central corpus callosum, and higher GMV in the bilateral middle temporal regions. Compared to controls, survivors scored lower for all cognitive domains, except for complex attention and cognitive flexibility. Amongst survivors, the GMV of the bilateral thalami and right ventral diencephalon correlated positively with working memory. **Conclusions**: Overall, PFT survivors demonstrated both alterations in GMV and cognitive functioning, with the most pronounced GMV deviations found in those treated with craniospinal radiation. Reduced GMV was associated with poorer cognitive performance.

## Simple Summary

Children who survive a posterior fossa tumor often require radiation therapy, which can be lifesaving but may also damage developing brain tissue. This treatment, along with the tumor and other therapies, can affect cognitive abilities such as memory, language, and attention. This study investigated whether survivors show differences in gray matter volumes and how these differences relate to cognitive abilities. By comparing anatomical magnetic resonance images and cognitive test scores from survivors with and without radiation therapy to controls, we identified specific brain regions with lower gray matter volume, particularly in survivors who received radiation therapy. Survivors also showed lower cognitive ability, and lower gray matter volume was related to lower working memory. These findings highlight the long-term impact of a brain tumor and its treatment on survivors and may help guide future clinical strategies to improve long-term outcomes.

## 1. Introduction

Two-thirds of pediatric brain tumors are posterior fossa tumors (PFT) [1,2]. PFTs, along with their treatment, and complications such as hydrocephalus or cerebellar mutism syndrome, may induce neurotoxicity and can adversely affect brain development. Induced neurotoxicity and altered brain development can result in cognitive impairment, which is one of the most debilitating long-term effects in survivors of pediatric brain tumors [3]. Research on cognitive outcomes reports persistent, and in some cases progressive, impairments. Yet long-term follow-up rarely extends beyond a few years, highlighting the need to monitor survivors during late adolescence and early adulthood [4,5,6].

Structural and functional neuroimaging techniques enable the evaluation of brain development and injury, enhancing our understanding of how pediatric brain tumors and their treatments relate to neural alterations and cognitive impairments [7,8,9]. Previous structural magnetic resonance (MR) imaging studies have shown that brain tumors and their treatment can impact both white and gray matter, with effects extending beyond the tumor region to widespread brain tissue [8,10,11,12]. Tumor-related complications, such as hydrocephalus, as well as several treatments such as chemotherapy or radiotherapy, can contribute to widespread brain damage [6]. Additionally, transneuronal degeneration of the cerebellar-subcortical-cortical pathways may further contribute to the observed widespread brain changes [13,14,15]. These pathways are critical for higher order cognitive functioning and require modulation of subcortical structures [13]. For example, white matter integrity of the thalamic–frontal pathway was correlated with volume loss in cerebellum and thalamus, while integrity of the superior longitudinal fasciculus (including the thalamus) was associated with verbal working memory [13,16].

While cortical gray matter is often less affected, subcortical and posterior fossa regions showed greater susceptibility to treatment-induced damage [17,18]. In particularly, mean radiotherapy dose and time elapsed since treatment are key predictors of subcortical gray matter volume loss, with the hippocampus showing the greatest vulnerability [19,20]. Similarly, survivors of adult-onset gliomas demonstrated widespread subcortical atrophy, with volumetric changes in hippocampi, /thalami, amygdalae, nuclei accumbens, pallida, and putamina, in a radiotherapy dose dependent manner [18,21].

Given the ongoing development of the pediatric brain and the potential progressive nature of gray matter volume loss, the evaluation of gray matter volumes in long-term survivors of pediatric PFT is an critical step to clarify the lasting impact of both the tumor and its treatment. Accordingly, this study aims to characterize cortical and subcortical gray matter volume alterations in survivors of PFT during late adolescence and early adulthood. Our objective was to detect regional as well as localized (voxel-wise) alterations in GMV. Additionally, we investigated whether these GMV alterations differed depending on the treatment received.

## 2. Materials and Methods

### 2.1. Participants

A total of 21 childhood PFT survivors treated at the University Hospitals Leuven were included. Included survivors were diagnosed with pilocytic astrocytoma (*n* = 8), ependymoma (*n* = 1), or medulloblastoma (*n* = 12). All survivors underwent a surgical resection of the tumor (partial resection *n* = 5 and complete resection *n* = 13). Fourteen survivors received additional photon radiotherapy (focal *n* = 2, craniospinal irradiation without *n* = 3, and with chemotherapy *n* = 9). None of the survivors were treated with chemotherapy as the sole adjuvant therapy. A detailed description of participant recruitment, informed consent, medical history, and population characteristics has been given previously [11]. Controls (*n* = 21, mean age 24.94 years, SD 4.76) were included and matched based on age, biological sex, and education. The ethical committee of the University Hospitals Leuven approved the study, all participants gave written informed consent before participation, and this study was conducted in accordance with the Helsinki Declaration.

### 2.2. Data Acquisition

#### 2.2.1. MR Neuroimaging

MR data were collected on a 3T Philips Achieva MR imaging scanner (Philips, Amsterdam, The Netherlands) with a 32-channel phased-array head coil. Imaging parameters for the T1-weighted anatomical images were as follows: repetition time/echo time = 4.6 ms/9.6 ms; 160 slices; voxel size = 0.98 mm × 0.98 mm; and slice thickness 1.2 mm.

T1-weighted images were visually inspected to ensure data quality. MR images for 3 survivors were excluded due to supratentorial lesions (non-irradiated survivor), extensive motion (irradiated survivor), or image artifacts (irradiated survivor). None of the controls were excluded.

#### 2.2.2. Cognitive Assessments

Participants were assessed with the Dutch abbreviated Wechsler Adult Intelligence Scale–Fourth Edition (WAIS-IV-NL) [22]. Estimates of intelligence subscales were acquired, i.e., full-scale intelligence quotient, perceptual reasoning index, verbal comprehension, processing speed, and working memory.

Additionally, several neuropsychological tests had been selected to assess key domains of cognitive functioning. Initially, test-specific z-scores were calculated based on the mean and standard deviation of performance scores of our own control population. Response time z-scores were sign-flipped to indicate better performance with higher z-scores across all tests. We then computed a domain-specific composite score by averaging the z-scores for tests classified under the corresponding DSM-5 cognitive domain [23]. An outline of these tests and their classification according to the cognitive domains is presented in Table 1. Cognitive assessments were successfully completed for all survivors and controls by a trained neuropsychologist (C.S. or E.T.) on the same day as the acquisition of the MR images.

### 2.3. Data Processing and Statistical Analyses

#### 2.3.1. MR Neuroimaging Processing

First, the T1-weighted MR images were bias field corrected using N4BiasFieldCorrection from Advanced Normalization Tools (ANTs) [26]. These images were further processed in the Computational Anatomy Toolbox version 12 (CAT12) for Statistical Parametric Mapping version 12 (SPM12), following the standard and recommended procedures described in the CAT12 manual [27]. Images were then normalized (standard MNI template) and segmented into gray matter, white matter, and cerebrospinal fluid, using tissue probability maps. Total intracranial volume was estimated for each participant. Segmented gray matter images were subsequently smoothed with a Gaussian kernel of 8 mm. Finally, we explicitly masked out the cerebellum and brain stem.

Second, the T1 weighted MR images were processed to perform region-based volumetric analysis using the FastSurfer pipeline. FastSurfer, a deep-learning based neuroimaging tool, generates cortical and subcortical parcellations [28]. Volumetric measures for each region were extracted from these parcellations using FreeSurfer’s ‘mri_segstats’ program. Subject-specific volumetric parameters were obtained for bilateral cortical regions defined according to the Desikan–Killiany–Tourville Atlas, for bilateral subcortical gray matter regions derived from the automated segmentation of deep gray matter structures, as well as for total supratentorial volume [29]. Cerebellar structures were excluded from all volumetric analyses.

To ensure the quality of the segmentations for both methods, segmentation results for all participants were visually quality-checked by researchers on the team (C.S., J.B., and K.B.). Examples of these segmentations can be found in Supplementary Materials (Figure S1).

#### 2.3.2. Statistical Analyses

Demographic variables, cognitive test scores, and domain-specific composite scores were compared between groups using Student’s *t*-test or non-parametric alternatives in the case of non-normal distributions. Demographic variables were compared between PFT survivors and controls, including biological sex, handedness, age at assessment, socioeconomic status, and self-reported Beck’s Depression Inventory. Socioeconomic status was calculated using a modified version of the Hollingshead Four Factor Index of Social Status [30]. Similar comparisons were conducted between irradiated and non-irradiated survivors, complemented with comparisons of survivor-specific variables such as age at diagnosis and time between diagnosis and study participation. One survivor who received only focal irradiation was excluded from the irradiated survivor subgroup for the between survivor analysis. All other irradiated survivors received craniospinal radiotherapy.

Voxel-based volumetry was conducted with a full factorial model in the SPM12 toolbox. Total intracranial volume was included as a covariate. The primary analysis compared contrasts in both directions between PFT survivors and controls, and the secondary analysis compared contrasts in both directions between irradiated and non-irradiated PFT survivors.

Region-based GMV was analysed using an ANCOVA adjusted for total supratentorial volume, with the same primary (PFT survivors vs. control) and secondary (irradiated vs. non-irradiated survivors) comparisons. False discovery rate (FDR) correction was applied to account for multiple comparisons.

Exploratory analyses examined associations between GMV and cognitive scores (full-scale intelligence quotient, verbal comprehension, perceptual reasoning index, working memory, processing speed, language, learning and memory, complex attention, and cognitive flexibility) within the survivor group for regions showing significant differences in GMV relative to controls. In the irradiated survivor group, GMV values were additionally correlated with age at radiotherapy, radiotherapy dose delivered to the whole brain (does not include any additional boosts to the PFT and/or tumor bed), and time since diagnosis. Pearson’s correlations or Spearman’s rank correlations were calculated, where applicable, and were FDR-corrected. All associative analyses were carried out in RStudio version 4.4.0.

## 3. Results

### 3.1. Group Characteristics

The survivor group did not significantly differ from the control group in terms of biological sex, handedness, age at assessment, or self-reported depression. While socioeconomic status differed between groups (p_unadjusted_ = 0.033), exploratory analysis including socioeconomic status as a covariate showed no significant effects and therefore is not reported further. The irradiated survivor group did not differ from the non-irradiated survivor group in terms of biological sex, handedness, age at assessment, self-reported depression, social economic status, age at diagnosis, age at assessment, or time between diagnosis and assessment. Detailed characteristics are shown in Table 2.

### 3.2. Gray Matter Volumetry

**Figure 1:**
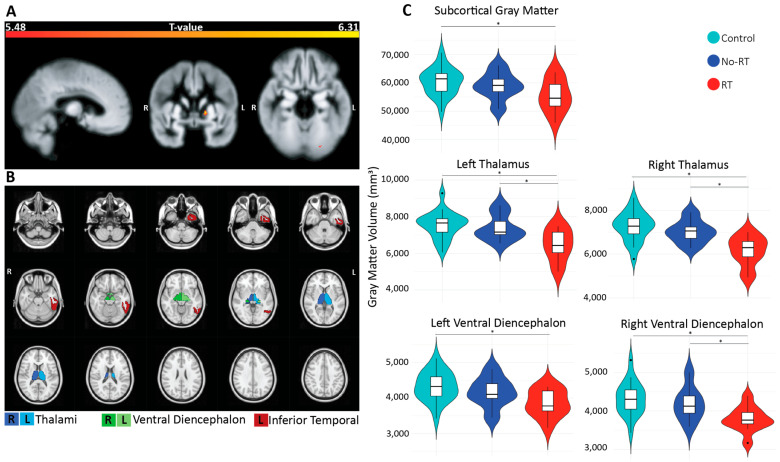
Group comparison results (survivors versus controls). This figure depicts (**A**) clusters with significantly lower voxel-based gray matter volumetry in survivors compared to controls across the entire supratentorial brain, (**B**) the cortical and subcortical regions with significantly lower (thalami and ventral diencephalon) or higher (inferior temporal) gray matter volumes in survivors than controls, and (**C**) for significant subcortical regions, violin plots were created showing the distribution of gray matter volume (mm^3^) along the *x*-axis. * for p_FDR_ < 0.05.

Based on the voxel-based analyses, significantly lower GMV was found for a cluster in the left occipital fusiform area (p_FWE_ = 0.03, k_E_ = 7) and in the left pallidum (p_FWE_ < 0.01, k_E_ = 37) in survivors compared to controls (see Figure 1A). No significant GMV differences were found between the irradiated and non-irradiated survivors. Based on the region-based analysis, subcortical regions showed significantly lower GMV for the total subcortical gray matter (p_FDR_ = 0.02), the bilateral thalami (left p_FDR_ < 0.01 and right p_FDR_ < 0.01), and the bilateral ventral diencephalon (left p_FDR_ < 0.01, right p_FDR_ < 0.01) (see Figure 1B,C). Greater GMV in survivors was found for the left inferior temporal region (p_FDR_ = 0.03).

Among irradiated and non-irradiated survivors, significantly lower GMV was observed in the irradiated survivors in the bilateral thalami (left p_FDR_ = 0.04 and right p_FDR_ = 0.03), right ventral diencephalon (p_FDR_ = 0.04), and central corpus callosum (p_FDR_ = 0.04).

Correlations between the GMV of regions with significantly lower GMV in survivors showed no significant association with radiotherapy dose delivered to the whole brain (left thalamus r = −0.31, *p* = 0.34; right thalamus r = −0.45, *p* = 0.14; right ventral diencephalon r = −0.33, *p* = 0.30), age at radiotherapy (range of r: −0.15–0.12, and *p* > 0.65), age at diagnosis (range of r: −0.13–0.13, and *p* > 0.69), nor with time since treatment (range of r: −0.07–0.27, and *p* > 0.40).

### 3.3. Cognitive Functioning and GMV Values

Survivors scored significantly lower (p_FDR_ < 0.001) for all WAIS-IV subscales: full-scale intelligence quotient, verbal comprehension, perceptual reasoning index, working memory, and processing speed. Survivors also scored significantly lower on composite scores for language (p_FDR_ < 0.01) and learning and memory (p_FDR_ < 0.01). No significant differences in cognitive outcomes emerged between survivor subgroups. However, visual inspection of the data indicate that irradiated survivors tended to score lower than non-irradiated survivors. Cognitive scores can be found in more detail in the Supplementary Materials (Table S1).

**Figure 2:**
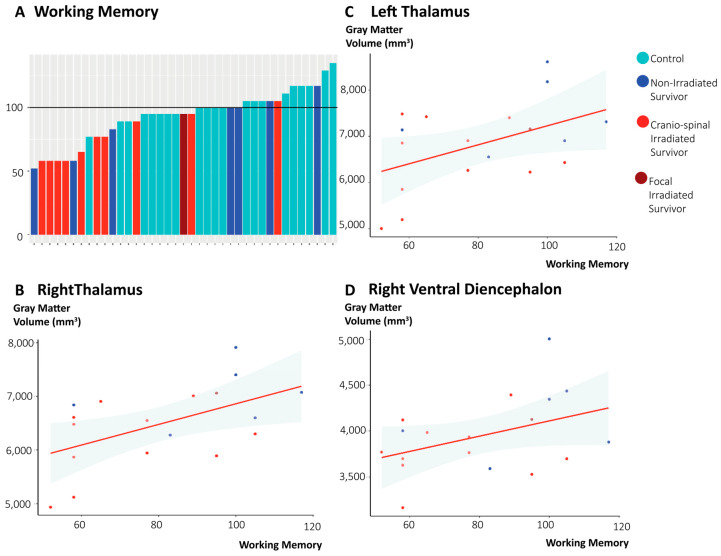
Cognitive functioning and the association with gray matter volumes. This figure depicts (**A**) a waterfall plot of the working memory scores from control (cyan), non-irradiated survivors (blue), focal irradiated survivors (burgundy), and craniospinal irradiated survivors (red). Other panels depict the significant (p_FDR_ > 0.05) relation of (**B**) the working memory scores and the gray matter volumes (mm^3^) of the left thalamus, (**C**) the right thalamus, and (**D**) the right ventral diencephalon.

Regarding associations between GMV and cognitive outcomes, positive correlations were observed between working memory scores and the GMV of the bilateral thalami (left thalamus: r = 0.49, p_FDR_ = 0.048; right thalamus: r = 0.50, p_FDR_ = 0.048), as well as the GMV of the right ventral diencephalon (r = 0.48, p_FDR_ = 0.048) (Figure 2). Remaining regions with significantly lower GMV in survivors were not significantly correlated with any of the cognitive scores.

## 4. Discussion

### 4.1. GMV Alterations

The present study evaluated cortical and subcortical GMV using both voxel-based and region-based volumetry in survivors of pediatric PFTs. Moreover, we explored potential associations between cognitive performance and GMV in long-term PFT survivors. We observed two small clusters of significantly reduced GMV in survivors compared to controls, located in the left pallidum and the left occipital fusiform gyrus. Beyond these focal differences, we observed several deep gray matter regions with reduced overall GMV. Furthermore, GMV alterations were not symmetrically distributed, potentially reflecting effects of the location of the tumor within the PFT or other influences such as handedness [31,32,33]. The discrepancies between the voxel-based and region-based analyses may reflect inherent methodological differences, but may also suggest that gray matter changes are subtle and more distributed across a region [34]. The divergence in results underscores the value of multi-modal imaging analyses to more comprehensively characterize morphological alterations in survivors of PFT [35,36].

Total subcortical GMV was lower in survivors compared to controls. This pattern extended to the bilateral thalami and ventral diencephalon, which were most pronounced in irradiated survivors. The GMV of the central corpus callosum differed only between irradiated and non-irradiated survivors, again with the lowest values for irradiated survivors. These results align with earlier reports of subcortical vulnerability of gray and white matter, including volume loss in the thalamus, red nucleus, putamen, and other subcortical areas [8,13,15,18,37]. In contrast, we did not replicate previously reported hippocampal GMV changes [18,20].

Although subcortical GMV was lower in our cohort, and lower whole-brain GMV was described in other studies, some cortical regions showed higher GMV in survivors relative to controls or in irradiated survivors compared to non-irradiated survivors. Such increases are not entirely unexpected, as greater GMV has been reported previously in the superior frontal gyrus of pediatric brain tumor survivors and in other neurodevelopmental disorders (i.e. attention-deficit hyperactivity disorder) [17,20,33,38]. Furthermore, the observed increase in GMV does not necessarily, reflect improved brain function [33]. Instead, it may be the result of altered or disrupted neurodevelopmental or neurobiological processes, including synaptic and neuronal pruning, gliosis, and neuroinflammation, which can arise from tumor pathology and treatment exposures and contribute to hypertrophic or dysregulated cortical patterns [20,39,40,41,42,43,44]. Another mechanism that can lead to widespread GMV alterations, is transneuronal degeneration, which could help explain the observed structural abnormalities observed even in non-irradiated survivors, who did not receive adjuvant treatment.

### 4.2. Cognitive Results and Relationship Between GMV

The PFT survivor group performed more poorly on all intelligence subscales, and in their composite scores of language and learning and memory relative to the control group. In contrast, we did not observe significant differences in composite scores for executive functioning and complex attention between survivors and controls. Although the literature suggests that irradiated survivors are at increased risk of cognitive impairment, our results did not reflect this anticipated difference compared to non-irradiated survivors [3].

Another important clinical risk of cognitive impairment in PFT survivors is cerebellar mutism syndrome, a spectrum of symptoms including, but not limited to, mutism [45,46]. In our retrospective review of medical records, mutism was reported in five PFT survivors, which may have contributed to the observed, albeit non-significant, differences in cognitive performance between irradiated and non-irradiated survivors. To assess the impact of cerebellar mutism syndrome on cognitive impairments, the full spectrum of symptoms should be evaluated using a standardized scoring scale, such as that proposed by Ricci et al. [47].

Despite the small group size and modest effect sizes, our results indicate that a lower GMV of the thalamus and the right ventral diencephalon was associated with worse working memory performance. This aligns with our previous work in this cohort, showing an association between structural network organization and working memory [11], and with studies showing that white matter integrity of the thalamic–frontal pathway relates to alterations in thalamic and cerebellar volume [13]. Further research is needed to elucidate the mechanisms by which these structural changes emerge following tumor occurrence and treatment, and to identify strategies for how to prevent it.

### 4.3. Limitations and Future Directions

The findings of this study must be interpreted in the context of some limitations. Pediatric PFTs have a low annual incidence and exhibit substantial population heterogeneity, resulting in a relatively small survivor population that includes individuals with varying tumor types, ages at diagnosis, and treatment regimens [48]. To maximize our cohort, we opted to include irradiated survivors who also had spinal metastasis (*n* = 5). Correcting for all subject-specific tumor- and treatment-associated adverse effects is not possible. Consequently, the results observed in our irradiated survivor group should be interpreted as potentially reflecting multiple contributing factors rather than a single causal effect. Broader collaborative efforts will be essential to increase cohort size and strengthen insights regarding the influence of specific risk factors, such as radiotherapy dose effects, cerebellar mutism, or age-related effects. Although group differences in GMV were observed, no voxel-level differences emerged between irradiated- and non-irradiated survivors. Moreover, in contrast to some previous studies, our results showed no differences in hippocampal GMV [18,20,49]. This discrepancy may be a consequence of methodological differences. Notably, our research focused on long-term survivors of pediatric PFT, who were on average 16.26 years post-treatment. By contrast, studies by Nagel et al. (2004) and Riggs et al. (2014) demonstrate decreased hippocampal growth shortly after diagnosis, with partial recovery after 2 years, and persistent lower GMV 5 years after diagnosis, compared to healthy controls [49,50]. Our data may suggest a more prolonged normalization or stable hippocampal GMV over time in long-term survivors.

We did not observe a significant association between the radiotherapy dose delivered to the whole brain and GMV, nor with any other clinical parameter. Our measurement of radiotherapy dose reflects the dose delivered to the whole brain, which may be too imprecise to detect dose-dependent volume decreases. In addition, the craniospinal radiotherapy-dose does not capture focal boosts to the clinical target volume, the dose distribution to the tumor, or the scatter of radiation to surrounding healthy tissue. However, prior research using region-specific radiotherapy dose estimates has demonstrated a dose-dependent GMV decrease in pediatric and adult brain tumor patients, as well as in pediatric leukemia patients [21,41,51]. Future research should consider to co-registering radiotherapy dose maps with GMV to study dose-dependency and a region’s susceptibility to radiotherapy-induced GMV alterations in more detail [52]. Additionally, longitudinal research is needed to track GMV trajectories across neurodevelopment and to clarify the age- and time-dependent effects of treatment, including both recovery processes and “growing into deficit” mechanisms related to time since treatment.

Finally, the composite domain scores were calculated based on a variety of cognitive tests. For composite scores of complex attention and executive functioning, we used different scores from the computerized Amsterdam’s Neuropsychological Tasks (ANT). Visual inspection of revealed substantial variability in these ANT measures at the subject-level, which may have affected the overall results. Nevertheless, including diverse measures allowed us to capture a broader range of attentional and executive processes, and is consistent with prior multimethod composite approaches.

## 5. Conclusions

In conclusion, both cortical and subcortical gray matter appear vulnerable to structural alterations following the occurrence and/or treatment of a posterior fossa tumor. Deep gray matter regions were particularly affected, with the thalamus and the ventral diencephalon showing lower GMV, especially among survivors who received radiotherapy during childhood. Additionally, GMV reductions in these regions were associated with working memory performance. Together, these findings underscore the importance of considering the effects of treatment on healthy-appearing gray matter, with particular attention to the heightened vulnerability of subcortical regions.

## Figures and Tables

**Table 1:** An outline of different key domains and cognitive test battery used.

Key Domain	Cognitive Assessments
Language	COWAT, Semantic fluency COWAT, Phonemic fluency PPVT
Learning and memory	Immediate free recall (total score trial 1–5), AVLT Long-term memory, AVLT Recognition recall, AVLT Immediate free recall (total score trial 1–5), RVDLT Long-term memory, RVDLT Recognition recall, RVDLT
Complex attention	Baseline speed, ANT: Reaction time and Stability Memory Search Letter, ANT: Reaction time and errors without distractor Shifting Attentional Set, ANT: Reaction time and errors in compatible condition
Cognitive flexibility	Memory Search Letter, ANT: Reaction time and error with 1 and 2 distractors Shifting Attentional Set, ANT: inhibition, errors in inhibition, cognitive flexibility, and errors of cognitive flexibility

COWAT: Controlled Oral Word Association Test [24]; PPVT: Peabody Picture Vocabulary Test; AVLT: Rey Auditory Verbal Learning Test; RVDLT: Rey Visual Design Learning Test; ANT: Amsterdam Neuropsychological Tasks [25].

**Table 2:** Demographic and medical characteristics of the PFT survivors and healthy controls included in the GMV analysis.

		Posterior Fossa Tumor Survivors			*p*-Value	
Parameter		Irradiated (*n* = 12)	Non-Irradiated (*n* = 6)	Healthy Controls (*n* = 21)	Survivors/ Control	Irradiated/ Non-Irradiated
						
Biological Sex	Male (N)	8	5	15	0.956	0.457
						
Handedness	Right (N)	11	6	17	0.209	0.467
						
Age at assessment	Years (M ± SD)	26.24 ± 5.01	23.85 ± 3.75	24.94 ± 4.76	0.571	
	Range	18.59–34.15	19.44–30.15	16.35–34.77		
						
Socioeconomic status	(M ± SD)	30.79 ± 14.69	28.33 ± 17.23	39.62 ± 11.25	0.033 *	0.772
	Range	9.00–58.00	8.00–47.50	19.00–60.50		
						
Beck’s Depression Inventory	(M ± SD)	7.25 ± 5.14	4.00 ± 5.90	5.76 ± 3.78	0.989	0.256
	Range	0.00–16.00	0.00–13.00	1.00–14.00		
						
Age at diagnosis	Years (M ± SD)	9.58 ± 4.60	6.12 ± 3.62			0.178
	Range	2.86–18.44	3.25–11.56			
						
Time between diagnosis and assessment	Years (M ± SD)	16.66 ± 12	17.11 ± 4.1			0.682
	Range	2.49–26.19	9.94–21.95			
						
Tumor Type	Pilocytic Astrocytoma	0	6			
	Medulloblastoma	11	0			
	Ependymoma	1	0			
						
Treatment type	Surgery Only	0	6			
	Chemotherapy	8	0			
	Focal Radiation	1	0			
	Craniospinal	11	0			
						
Radiotherapy dose ^1^		34.00 ± 5.56				
		23.4–40.0				
Hydrocephalus	Yes (N) with shunt (N)	11 6	2 2			
Mutism ^2^	Yes (N)	4	1			
						

**Note:** Values are presented as mean ± standard deviation for the following variables: age at assessment, socioeconomic status, Beck’s Depression Inventory score, age at diagnosis, and time between diagnosis and assessment. *p*-values reflect results from independent-samples *t*-tests or non-parametric alternatives (Mann–Whitney U test or chi-square test) when appropriate. ^1^ Radiotherapy dose refers to the dose delivered to the whole brain and does not include any additional boosts to the PFT and/or tumor bed. ^2^ As reported in the medical files through clinical observation. Statistically significant group differences (*p* < 0.05) are indicated with an asterisk (*).

## Data Availability

The data for this study are available from the corresponding author upon reasonable request.

## Supplementary Materials

The following supporting information can be downloaded at https://www.mdpi.com/article/10.3390/cancers18030495/s1, Figure S1: Segmentation examples of FastSurfer; Table S1: Overview of neurocognitive functioning.

## Abbreviations

The following abbreviations are used in this manuscript:

AVLT	Rey Auditory Verbal Learning Test
COWAT	Controlled Oral Word Association Test
DSM-5	Diagnostic and Statistical Manual of Mental Disorders
FDR	False Discovery Rate
FEW	Family-Wise Error
GMV	Gray Matter Volume
MR	Magnetic Resonance
PFTs	Posterior Fossa Tumors
PPVT	Peabody Picture Vocabulary Test
RVDLT	Rey Visual Design Learning Test
SD	Standard Deviation
SPM12	Statistic Parametric Mapping
WAIS-IV-NL	Wechsler Adult Intelligence Scale–Fourth Edition–Dutch
