# Supplementary material for: Gray Matter Volumetry and Cognitive Functioning in Pediatric Posterior Fossa Tumor Survivors
Source: Cancers (Basel). 2026 Feb 2;18(3):495. doi: 10.3390/cancers18030495 (PMC12897364; doi:10.3390/cancers18030495)
Supplement: Supplementary file 1 [file cancers-18-00495-s001.zip › cancers-4063667-supplementary.pdf]

Supplementary Materials

Figure S1. Segmentation examples of FastSurfer

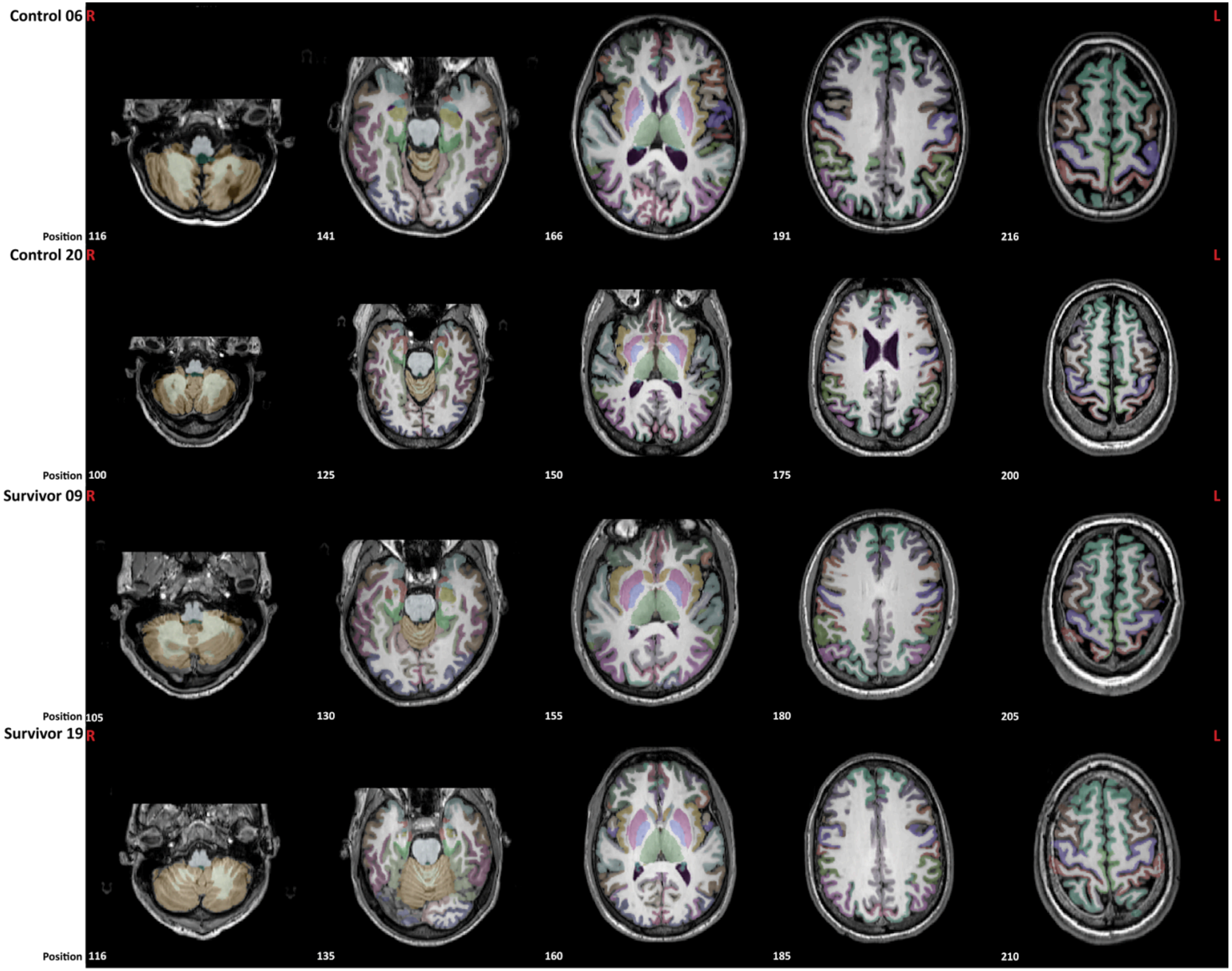

Table S1. Overview of neurocognitive functioning

	Control	Posterior Fossa Tumor Survivor	
	n=21	n=18	PFDR
Total Intelligence	106.76±14.92	79.94±13.05	<0.001
	80 - 130	59 - 105	
Verbal Comprehension	107.67±16.83	84.22±16.99	<0.001
	68 - 140	51 - 122	
Perceptual Reasoning	107.05±14.72	86.89±13.14	<0.001
	75 - 129	62 - 110	
Processing Speed	101.9±14.05	78.83±13.36	<0.001
	73 - 125	52 - 97	
Working Memory	103.38±13.76	80.56 ± 20.86	<0.001
	77- 135	52 - 117	
Domain Scores			
Language	0±0.76	-0.69±0.67	0.006
	-1.09 - 1.5	-1.74 - 0.81	
Learning and Memory	0±0.83	-0.87±0.95	0.006
	-2.81 - 1.02	-3.27 - 0.51	
Complex Attention	0.04-0.6	-0.36±0.94	0.133
	-1.78 - 0.84	-3.26 - 0.8	
Executive Functioning	0.05±0.57	-0.31±0.83	0.133
	-1.31 - 0.88	-2.03 - 0.99	

Note: Mean ± Standard deviations
